# Systematic review of clinical practice guidelines for acne vulgaris published between January 2017 and July 2021

**DOI:** 10.1002/ski2.240

**Published:** 2023-05-23

**Authors:** Louise Corcoran, Ingrid Muller, Alison M. Layton, Gwennan Rucinski, Viktoria Venkatess, Anicka Sufraz, Sophie Dove, Mark Lown, Beth Stuart, Nick Francis, Miriam Santer

**Affiliations:** ^1^ University of Southampton Southampton UK; ^2^ Skin Research Centre Hull York Medical School University of York York UK; ^3^ Salisbury NHS Foundation Trust Salisbury UK; ^4^ Public Contributor Southampton UK; ^5^ Queen Mary University of London London UK

## Abstract

**Background:**

Acne is very common, can cause considerable negative impact on quality of life and there is increasing concern over the use of long courses of oral antibiotics for this condition.

**Objectives:**

(1) To critically appraise reporting in acne guidelines and compare this with previous systematic review of acne guidelines. (2) Examine acne treatment guidance on pre‐specified acne treatments of interest and compare between acne guidelines.

**Methods:**

Searches for new or updated guidelines were carried out in MEDLINE, Embase, Google Scholar, LILACS from 1 January 2017 to 31 July 2021, supplemented by searching a guideline‐specific depository and checking for updates to guidelines included in previous review. We included guidelines, consensus statements or care protocols on the medical treatment of acne vulgaris in adults and/or children and excluded those that focused on a single intervention or subgroup of acne, regional adaptations of guidelines or guidelines included in previous review. AGREE II checklist was applied to critically appraise reporting of guidelines. Results were synthesised narratively.

**Results:**

Of 807 abstracts identified nine guidelines were identified that were eligible for inclusion. All guidelines had AGREE II scores above average in at least one domain and reporting was substantially improved compared to the systematic review of acne carried out 5 years previously. There was consensus between guidelines on the key role of topical treatments as first‐line acne treatment and most recommended continuing topical treatments as maintenance therapy. There was considerable variation between guidelines on classification of severity, indications for commencing oral antibiotics and on maximum duration of oral antibiotics. However, there was consensus on the need for co‐prescription of a non‐antibiotic topical treatment when using oral antibiotics. There were notable differences on recommendations regarding provision of information for patients on how to use topical treatments or how to mitigate against side effects.

**Conclusions:**

Substantial differences in classification of acne severity hampered comparisons between guidelines. Although development and reporting of guidelines has improved over the past 5 years, differences in key recommendations remain, possibly reflecting uncertainties in the underlying evidence base. Differences between guidelines could have substantial implications for prevalence of antibiotic prescribing for acne.

## Graphical abstract

This systematic review of acne guidelines highlights the considerable variation between guidelines on classification of acne severity, indications for commencing oral antibiotics and on maximum duration of oral antibiotics. Although development and reporting of guidelines has improved over the past 5 years, differences in key recommendations remain, possibly reflecting uncertainties in the underlying evidence base.

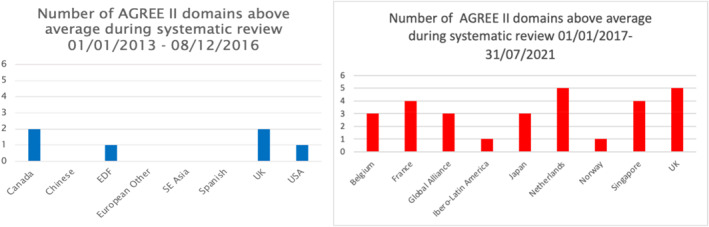

1

**What is already known about this topic?**

- Acne treatment guidelines have a key role in ensuring optimal treatment while minimising antibiotic burden and potential antibiotic resistance.
- Previous reviews of acne treatment guidelines highlighted variable quality and low scores on critical appraisal.

**What does this study add?**

- New guidelines and updated guidelines score more highly on critical appraisal criteria than five years ago.
- Key differences between guidelines remain on several points, including around the prescribing of long courses of oral antibiotics.

## 2 BACKGROUND

Acne vulgaris (hereon referred to as acne) is one of the most prevalent global skin conditions and causes substantial burden in terms of physical and psychosocial wellbeing and costs of management.^1^ There is rising concern about the widespread use of antibiotics for acne and impact on antibiotic resistance.^2^ Acne treatment guidelines have a key role in ensuring optimal treatment for the condition while also minimising antibiotic burden and potential antibiotic resistance.

Previous reviews of acne treatment guidelines highlighted variable quality, low scores on trustworthiness and lack of stakeholder engagement.^3^, ^4^, ^5^ The most recent of these reviews was published in 2017. Several acne treatment guidelines have been published since that time, so we aimed to update this review and compare quality by re‐appraising using the AGREE II checklist.^6^ Furthermore, we aimed to compare the degree of agreement on key aspects of acne management between guidelines.

## 3 METHODS

Our research methods were designed to meet the following aims:

- To update the previous systematic review of acne clinical practice guidelines
- To use AGREE II checklist to critically appraise reporting in acne guidelines
- To examine guidance on pre‐specified acne treatments of interest

The protocol for this review was published prospectively on PROSPERO CRD42021269296.

### 3.1 Data sources and guideline selection

Searches for new or updated guidelines were carried out in the following databases: MEDLINE, Embase, Google Scholar, LILACS applying search dates from 1 January 2017 to 31 July 2021 in order to update the previous systematic review.^3^ Search criteria shown in Box 1. We supplemented this with searching a guideline‐specific depository^7^ and by checking for updates to guidelines included in previous review.^3^

**BOX 1 Search strategy**

**Table:** 

Source	
Databases	Search terms used
MEDLINE	Acne (in title or abstract) OR acne vulgaris (mapped to the thesaurus) AND any one of the terms: Guideline, algorithm or recommend* (in title or abstract)
Embase	Acne (in title or abstract) OR acne vulgaris (mapped to the thesaurus) AND any one of the terms: Guideline, algorithm or recommend* (in title or abstract)
LILACS	Acne AND any of guideline, algorithm or recommend*

We included guidelines, consensus statements or care protocols on the medical treatment of acne vulgaris in adults and/or children that included a range of treatments. We excluded guidelines that focused on a single intervention or on a subgroup of acne, such as severe acne, truncal acne, or acne maintenance or other specific patient group, such as skin of colour. We excluded conference abstracts, editorials, letters, regional adaptations of guidelines or guidelines that were included in a previous systematic review. We included only the most recent update of any particular guideline. No language restrictions were applied.

Two reviewers (GR and VV) scanned titles and abstracts for papers meeting the inclusion criteria; any disagreements were resolved by discussion with a third reviewer (MS). Where necessary, full text articles were obtained in order to assess whether inclusion criteria were met. Any guidelines not in English were translated prior to assessment and data extraction.

### 3.2 Guideline quality assessment

We carried out a critical appraisal of guideline quality, using the Appraisal of Guidelines for Research and Evaluation (AGREE) II Reporting Checklist (AGREE II).^6^ The AGREE II checklist, as shown in Box 2, comprises 23 items in six domains with each item scored on a scale from 1 to 7 (1 = strongly disagree; 7 = strongly agree).

**BOX 2 AGREE II Reporting Checklist**

**Table:** 

AGREE II domains	Key items
1. Scope and purpose	The overall objective(s) of the guideline is (are) specifically described. The health question(s) covered by the guideline is (are) specifically described. The population (patients, public, etc.) to whom the guideline is meant to apply is specifically described.
2. Stakeholder involvement	4. The guideline development group includes individuals from all relevant professional groups. 5. The views and preferences of the target population (patients, public, etc.) have been sought. 6. The target users of the guideline are clearly defined.
3. Rigour of development	7. Systematic methods were used to search for evidence. 8. The criteria for selecting the evidence are clearly described. 9. The strengths and limitations of the body of evidence are clearly described. 10. The methods for formulating the recommendations are clearly described. 11. The health benefits, side effects, and risks have been considered in formulating the recommendations. 12. There is an explicit link between the recommendations and the supporting evidence. 13. The guideline has been externally reviewed by experts prior to its publication. 14. A procedure for updating the guideline is provided.
4. Clarity of presentation	15. The recommendations are specific and unambiguous. 16. The different options for management of the condition or health issue are clearly presented. 17. Key recommendations are easily identifiable.
5. Applicability	18. The guideline describes facilitators and barriers to its application. 19. The guideline provides advice and/or tools on how the recommendations can be put into practice. 20. The potential resource implications of applying the recommendations have been considered. 21. The guideline presents monitoring and/or auditing criteria
6. Editorial independance	22. The views of the funding body have not influenced the content of the guideline. 23. Competing interests of guideline development group members have been recorded and addressed.

In the final section of the AGREE II checklist the overall guideline assessment is carried out. Box 3 illustrates the two questions.

**BOX 3 Overall Guideline Assessment**

**Table:** 

Question	Scoring options
1. Rate the overall quality of this guideline.	**7‐point scale (1 is lowest possible quality, 7 is highest possible quality)**
2. I would recommend this guideline for use.	**Yes**
	**Yes, with modifications**
	**No**

Information relevant to the AGREE II checklist was extracted from included guidelines by two reviewers (GR/LC or VV/AS) and scores collated in the online platform. Discrepancies in scores were discussed and resolved with a third reviewer (MS) where necessary. To allow comparison with previous systematic review of acne guidelines,^3^ we reported scores using a 5‐point Likert scale as shown in Box 4.

**BOX 4 5‐point Likert scale**

**Table:** 

Percentage (%) score	Rating
Over 80	Excellent
Over 60 and up to 80	Good
Over 40 and up to 60	Average
Over 20 and up to 40	Fair
20 or under	Poor

### 3.3 Guideline recommendation extraction

We extracted recommendations from all guidelines that met inclusion criteria regardless of quality score. Data on recommendations on pre‐specified treatments of interest were extracted into a pre‐piloted spreadsheet by two independent reviewers (GR/LC or VV/AS) with discrepancies resolved with a third reviewer (MS) where necessary.

Where available, the following treatments of interest were extracted:

1. First line treatment for acne
2. Second line treatment for acne
3. Third line treatment for acne
4. Guidance on oral antibiotics
  - When to start
  - Duration of treatment
  - Co‐prescribing
5. Guidance on isotretinoin
  - Requirements prior to referral/treatment
  - Who can prescribe isotretinoin?
6. Dietary guidance
7. Guidance about providing information for patients on how to use topical treatments and mitigating against side effects

No ethical approval was sought, as all data are publicly available.

## 4 RESULTS

**FIGURE 1:**
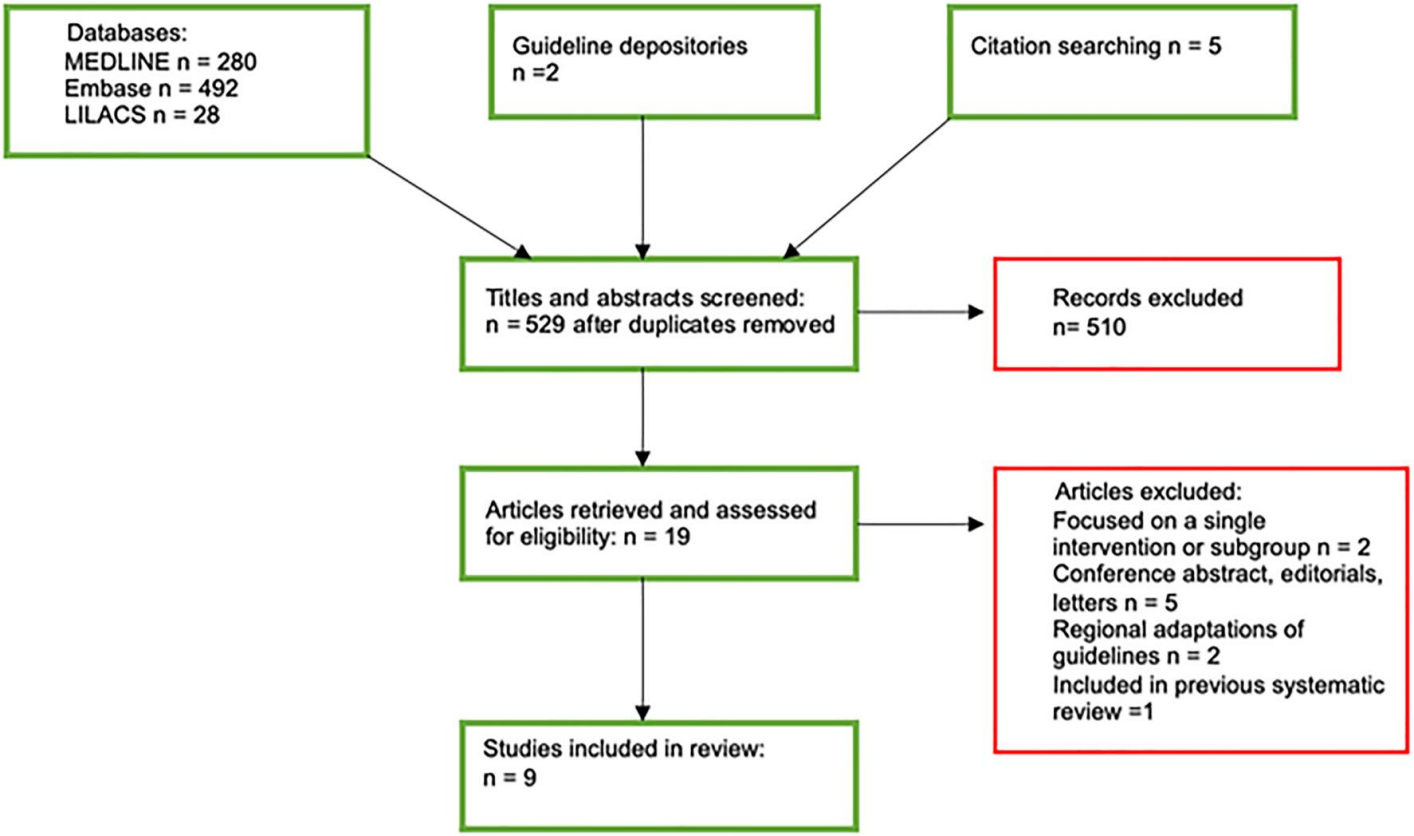
PRISMA flowchart.

The search retrieved 807 titles, of which 19 were identified as potentially eligible after removing duplicates and screening the title and abstract. Following this process, the full‐text articles were further assessed, and 10 articles were removed due to not meeting the inclusion criteria. There were nine guidelines that were included in this systematic review, and this pathway is illustrated in Figure 1.

Of the nine guidelines fulfilling the eligibility criteria, three required translation, which was carried out using online software^8^ and checked with a native speaker.

### 4.1 Guideline quality assessment – Rigor scores

**FIGURE 2:**
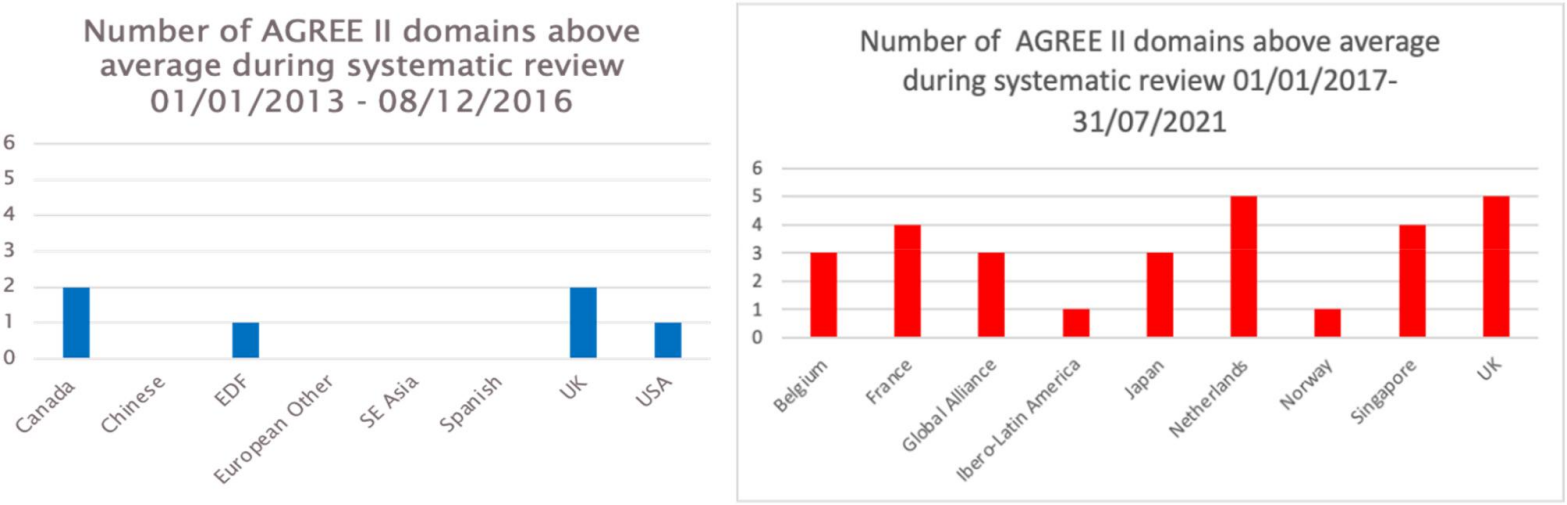
AGREE II domains above average.

The characteristics of included guidelines are shown in Table 1. All guidelines had AGREE II scores above average in at least one domain. The overall quality assessment rating of the guidelines were in the range of 2.5‐6 (Table 2). It was also identified that the AGREE II scores were substantially improved compared to the previous systematic review of acne carried out (Figure 2).

**TABLE 1:** Summary of adjusted scores using the AGREE II reporting checklist.

Guideline	1.Scope and purpose	2.Stakeholder involvement	3.Rigour of development	4.Clarity of presentation	5.Applicability	6.Editorial independence	Number of domains above average (using 5‐point likert scale)
Belgium^9^	94	56	57	64	21	83	3
France^10^	92	44	74	100	13	83	4
Global Alliance^11^	81	64	52	86	46	42	3
Ibero‐Latin American^12^	56	22	22	67	17	46	1
Japan^13^	78	19	58	75	25	75	3
Netherlands^14^	83	92	81	89	54	71	5
Norway^15^	19	17	25	69	6	21	1
Singapore^16^	92	61	52	81	17	75	4
UK^17^	92	92	91	94	69	50	5

*Note*: Excellent (over 80%) in green, good (over 60%–80%) in green, average (over 40%–60%) in orange, fair (over 20%–40%) in orange and poor (20% or under) in red.

**TABLE 2:** Summary of Overall Quality Assessment Rating using the AGREE II reporting checklist.

Guideline	Rate the overall quality of this guideline	I would recommend this guideline for use
Belgium^9^	5/67%	Yes
France^10^	5.5/75%	Yes + modifications
Global Alliance^11^	5.5/75%	Yes
Ibero‐Latin American^12^	2.5/25%	No
Japan^13^	5/67%	Yes + modifications
Netherlands^14^	6/83%	Yes
Norway^15^	4.5/58%	Yes + modifications
Singapore^16^	5/67%	Yes + modifications
UK^17^	6/83%	Yes

### 4.2 Guideline recommendations on key pre‐specified treatments of interest

Key recommendations from the nine included guidelines on treatments of interest are summarised in Table S1.

#### 4.2.1 First and second‐line treatment recommendations for acne

Comparisons of guidelines on recommendations regarding first and second‐line treatments was challenging as the guidelines varied in their approach to classification of acne, and therefore in their treatment recommendations. Where some guidelines use a mild/moderate/severe acne classification, with related treatment recommendations, others differentiated treatment pathways depending on classifications such as: “comedonal”, “papulopustular”, “inflammation and comedones.” Not all guidelines framed management recommendations in terms of specifying first, second, and third line treatments for acne. It was therefore decided to adjust the approach to consider only first and second line treatments. All guidelines recommended either topical benzoyl peroxide or topical retinoid (mainly adapalene) as first line treatments, but with marked differences regarding whether these were to be prescribed individually or in combination with each other or with another agent (e.g. topical clindamycin, azelaic acid or with oral antibiotics) (Table S1).

#### 4.2.2 Indications for commencing oral antibiotics

The recommendations regarding when to start oral antibiotics varied between guidelines. In five guidelines oral antibiotics were recommended for moderate or severe acne,^11^, ^12^, ^14^, ^16^, ^17^ although one guideline suggested oral antibiotics for mild or moderate acne.^10^ In one guideline it was advised that oral antibiotics should be started only in severe papulopustular acne,^9^ but another guideline suggested initiation of oral antibiotics if topical treatments had been insufficient or if the truncal area was involved.^11^ One guideline suggested oral antibiotics should be commenced for “inflammatory acne”^13^ (Table S1).

#### 4.2.3 Duration of treatment with oral antibiotics

All guidelines were consistent in recommending limiting the duration of treatment with oral antibiotics for acne. However, the maximum duration of oral antibiotic treatment varied from 6 weeks to 6 months, although only one guideline^17^ suggested treatment could be continued to 6 months (Table S1).

#### 4.2.4 Co‐prescribing with oral antibiotics and maintenance treatment following oral antibiotics

All guidelines emphasised the importance of co‐prescribing non‐antibiotic topical acne treatments alongside oral antibiotics. This is important to reduce the potential development of antibiotic resistance by improving efficacy (and therefore need for exposure to antibiotics) or by directly reducing both sensitive and resistant strains of Cutibacteria acnes. All guidelines included information on the use of topical treatments as maintenance therapy to prevent recurrence of acne after discontinuing oral antibiotics. Seven guidelines suggested this should be offered routinely, although the remaining two guidelines^15^, ^17^ suggested that this should be considered in some circumstances.

#### 4.2.5 Recommendations on isotretinoin for acne

Dessinioti et al.^18^ have published a comparison of guidelines and consensus articles on the management of acne with oral isotretinoin. They address the question regarding indications for treatment with oral isotretinoin with acne, but did not examine the question regarding who can prescribe oral isotretinoin, which varies between guidelines. Only four of the guidelines made reference to who can prescribe isotretinoin, but in some it was unclear (e.g. “mainly a dermatologist/specialist in skin diseases”). Two guidelines suggested isotretinoin prescription should be carried out by a dermatologist only,^15^, ^16^ although another two guidelines recommended that this could be carried out by a dermatologist or by a GP^9^, ^14^ (Table S1).

#### 4.2.6 Dietary guidance

In one guideline no information was given on dietary recommendations.^12^ The remaining eight guidelines mentioned diet, but only four gave specific recommendations such as “low glycaemic index diet”^9^, ^14^, ^16^, ^17^ or avoiding certain foods, such as chocolate, whey or milk.^16^ Two guidelines made reference to the importance of promoting a healthy, balanced diet and considering the risks of the development of eating disorders when giving dietary recommendations^13^, ^17^ (Table S1).

#### 4.2.7 Recommendations regarding provision of information for patients on how to use topical treatments and mitigating against side effects

Measures for reducing side effects of topical treatments were discussed in most guidelines, but they differed in what advice they recommended. Seven guidelines recommended starting topical agents with less frequent application or limiting the duration of application.^9^, ^10^, ^11^, ^14^, ^15^, ^16^, ^17^ Three guidelines advised informing patients to use emollients to reduce dry skin.^10^, ^12^, ^14^ Only two guidelines recommended providing information to patients on the need to continue regular treatment for several weeks in order to see effects.^10^, ^15^ One guideline made no mention of advice to give patients regarding reducing side effects from topical treatments.^13^

## 5 DISCUSSION

We identified nine new clinical guidelines on acne that had been published since 2016 and found that many aspects of acne guideline development and reporting have improved, particularly transparency around conflicts of interest and inclusion of patients, methodologists and all target users (e.g. generalists) on guideline development groups. In other respects there seems to be slower progress, such as equal consideration to benefits and harms of included interventions to ensure recommendations take account of both.

This review highlights the consistency in guidelines in some recommendations, such as the need to co‐prescribe topical treatments alongside oral antibiotics, but also more subtle variations between guidelines in whether to continue maintenance therapy with topical treatments after discontinuing antibiotics. There were notable differences relating to use of oral antibiotics, for instance around indications for commencing therapy and maximum duration of therapy. There were also marked differences in some very common prescribing questions, such as whether to commence first line treatment with monotherapy or combination topical treatments, which may reflect gaps in the evidence base on acne treatments. Despite increasing international awareness of the need for antibiotic stewardship in acne, the differences in access to non‐antibiotic treatments is striking, such as oral isotretinoin, due to professional barriers in who is able to prescribe this.

### 5.1 Findings in context of existing research

A comparison of guidelines on the management of acne with oral isotretinoin similarly noted that comparisons across guidelines are limited by the use of different classification systems for grading acne severity.^18^

### 5.2 Strengths and limitations

We increased the scope of previous systematic reviews on acne guidelines by extracting data and comparing recommendations from the guidelines identified in order to highlight differences, particularly as they relate to recommendations relevant to limiting the use of long‐term oral antibiotics. However, there are limitations in this approach due to the variations in classification of grading acne severity. Furthermore, we were unable to compare the differing recommendations regarding use of hormonal treatments, such as combined oral contraceptive, co‐cyprindiol and spironolactone. We fully acknowledge that this selection of guidelines reviews reflects a ‘snapshot’ in the constant evolution of guideline development.

We did not extract data from guidelines on recommendations regarding assessment of mental health impact of acne, or on measurement of patient‐reported outcome measures in acne, or on the methods used for patient involvement in guideline development, all of which would have strengthened this review.

### 5.3 Implications for research

The previous review on this topic noted that, *“Although only a few classes of drug are used to treat acne vulgaris, they can be prescribed in numerous different two or three‐way combinations, most of which have never been compared in randomised controlled trials. This results in significant evidence gaps and makes formulating any comprehensive guideline for acne difficult”.* Although there are still very few direct comparisons, an increasing number of systematic reviews with network meta‐analysis have directly or indirectly informed more recent guidelines,^17^, ^19^ although the heterogeneity of outcome assessment and differing classifications of severity between the different RCTs within the reviews may have hampered their findings. Harmonising outcome measures and classifications in acne is an urgent requirement in order to properly inform treatment recommendations.^20^, ^21^

Although some differences between guideline recommendations are likely to arise from methodological differences in underpinning research, in other areas the discrepancies in advice reflect a lack of evidence. For instance, there is only slim evidence on which to base recommendations regarding dietary advice for acne,^22^ the need for maintenance treatment and what specific advice is most necessary in order to mitigate against side effects of common treatments.

### 5.4 Implications for practice

There is a clear role for topicals, and the provision of sufficient advice on how to use these is important. Given topical agents require treatment for several weeks, this may suggest that signposting patients towards high quality evidence‐based advice to support treatment adherence. There is a need for increased emphasis and consensus on the risks of antibiotic resistance with oral antibiotics. This systematic review of acne guidelines has indicated that access to oral isotretinoin varies. Therefore, it may be helpful to consider how people with acne that meet the licensed indications for isotretinoin secure their treatment in their care pathway.

## CONFLICT OF INTEREST STATEMENT

Alison M. Layton has provided unrestricted educational talks or acted as a consultant on research developments for Proctor & Gamble, Galderma Pharmaceuticals, La Roche‐Posay, Leo Pharma, Novartis, L’Oreal, Beiersdorf and Origimm. She is currently a member of the British Association of Dermatologists Retinoid Working Group. She chairs a National UK Acne Group and is a member of the Personalising Acne Consensus of Experts which has superseded the Acne Global Alliance implemented to improve outcomes in acne management. The latter 2 groups are supported by Galderma Pharmaceuticals. The other authors declare that they have no known conflicts of interest.

## AUTHOR CONTRIBUTIONS

**Louise Corcoran**: conceptualization (equal); data curation (equal); investigation (equal); writing—original draft (lead); writing—review & editing (lead). **Ingrid Muller**: conceptualization (equal); writing—review & editing (equal). **Alison M. Layton**: conceptualization (equal); writing—review & editing (equal). **Gwennan Rucinski**: conceptualization (equal); data curation (equal); investigation (equal); writing—review & editing (equal). **Viktoria Venkatess**: conceptualization (equal); data curation (equal); investigation (equal); writing—review & editing (equal). **Anicka Sufraz**: conceptualization (equal); data curation (equal); investigation (equal); writing—review & editing (equal). **Sophie Dove**: writing—review & editing (equal). **Mark Lown**: conceptualization (equal); writing—review & editing (equal). **Beth Stuart**: conceptualization (equal); methodology (lead); writing—review & editing (equal). **Nick Francis**: conceptualization (equal); writing—review & editing (equal). **Miriam Santer**: conceptualization (equal); data curation (equal); investigation (equal); writing—original draft (lead); writing—review & editing (lead).

## ETHICS STATEMENT

Not applicable.

## Supporting information

Table S1

## Data Availability

Data derived from public domain resources.
