# Supplementary material for: Systematic review of clinical practice guidelines for acne vulgaris published between January 2017 and July 2021
Source: Skin Health Dis. 2023 May 23;3(4):e240. doi: 10.1002/ski2.240 (PMC10395621; doi:10.1002/ski2.240)
Supplement: Supplementary file 1 — Table S1 [file SKI2-3-e240-s001.docx]

**APPENDIX**

**Table S1 Guideline recommendations on acne treatment**

| Guideline | First line treatment for mild/moderate acne | Second line treatment for mild/moderate acne | Oral antibiotics – indications for initiation | Oral antibiotics – duration of treatment | Oral antibiotics – prescribing topicals after stopping | Isotretinoin - who can prescribe? | Dietary guidance | Information on how to use topical treatments and mitigating against side effects |
| --- | --- | --- | --- | --- | --- | --- | --- | --- |
| Belgium^9^ | **Comedonal acne**: topical BPO, topical retinoid (adapalene) or azelaic acid  **Mild to moderate papulopustular acne:** topical combination product | **Comedonal acne**: topical combination product (from first line treatment) | Severe papulopustular acne | 4 months | Maintenance therapy with adapalene for mild to moderate acne, and for severe forms of acne use adapalene and BPO | GP or dermatologist | - Consider avoiding food with a high GI. Some experts suggest avoiding dairy products, but insufficient evidence. | - BPO is equally effective at lower concentrations with fewer side effects - Consider adjusting frequency of application and progressively increasing dosing |
| France^10^ | **Almost clear/almost no lesion**: BPO OR topical retinoid  **Mild**: BPO and topical retinoid  **Moderate**: BPO and topical retinoid OR oral doxycycline or lymecycline and BPO and topical retinoid | **Almost clear/almost no lesion**: BPO and topical retinoid  **Mild**: Intensify first-line topical OR topical treatment with antibiotic and retinoid or azelaic acid OR oral doxycycline or lymecycline and BPO and topical retinoid  **Moderate**: Oral isotretinoin | Second line treatment for mild acne, can be first line for moderate acne | 3 months | Maintenance treatment with tretinoin or adapalene or BPO | No information given | - No dietary restrictions recommended | - Continue use over several weeks required for improvement - Prevent irritation by applications on alternate days and using moisturisers. |
| Global Alliance^11^ | **Mild acne (comedonal)**: topical retinoid or fixed combination with retinoid  **Mild papular/ pustular**: fixed combination OR BPO OR topical retinoid OR azelaic acid. **Moderate papular/ pustular**: fixed combination preferred AND/OR hormonal therapy AND/OR oral antibiotic. | Fixed combination preferred AND/ OR hormonal therapy AND/OR oral antibiotic | Inflammatory acne not responding to topical treatments; truncal acne or multiple body areas; moderate- to moderately severe acne | 3-4 months | Maintenance advised is topical retinoids with or without BPO OR azelaic acid | No information given | - Improving insulin   resistance through diet and lifestyle can improve acne | - Discuss mild irritation can occur but usually subsides after 1-2 weeks - Advise using a small dose (FTU/ pea size) - A gentle cleansing programme recommended, but avoid over-cleansing - Choose a retinoid formulation appropriate for climate and season, titrate dose at initiation |
| Ibero-Latin American^12^ | **For mild papulopustular acne***: fixed combination (BPO and clindamycin OR BPO and adapalene OR BPO and erythromycin OR tretinoin and clindamycin) OR 'antimicrobials' (BPO or azelaic or clindamycin or dapsone) OR retinoids (tretinoin or adapalene or tazaratotene) | No specifications of first or second line treatments for topical agents | Moderate or severe papulopustular acne, but severity categorisations are not defined | 6 – 12 weeks | Maintenance treatment with BPO (1st line) or azelaic acid (2nd line) | No information given | - No information given | - Use of moisturisers and photoprotectors can reduce the risk of photosensitivity and post- inflammatory pigmentation |
| Japan^13^ | **Mild inflammation & comedones*:** Fixed combinations of: clindamycin and BPO OR adapalene and BPO OR adapalene and topical antimicrobial. Alternatively monotherapy with: BPO OR adapalene OR topical antimicrobial  **Moderate inflammation & comedones***: similar to mild inflammation and comedones, but can also include combinations with oral antimicrobials | First and second line for mild, moderate and severe acne not differentiated. | “Treatment of inflammatory acne lesion” | 6 weeks - 3 months | Maintenance is adapalene or BPO or adapalene/ BPO | No information given | - “We recommend against deviated food habits and maintaining a well-balanced diet." | No information given |
| Netherlands^14^ | **Mild acne**: topical BPO OR tretinoin OR adapalene  **Moderate acne**: same as mild acne plus topical clindamycin or erythromycin. | **Mild acne**: same as first line for moderate acne. If comedones only then BPO with either adapalene or tretinoin. **Moderate acne**: same as first line for severe acne. If insufficient effect then consider oral isotretinoin. | Moderate acne not responding to topicals; first line treatment in severe acne. | 6 weeks - 3 months | Maintenance with topical retinoid or BPO | GP or dermatologist | - “No convincing evidence for a relationship between specific foods (e.g. chocolate, dairy products, pork, fatty foods) and onset or worsening of acne”. - Foods with a high GI possibly aggravate acne | - Advice to apply BPO and topical retinoids at night, and topical antibiotics in morning - Consider use of an emollient for dry skin - Initially use a low concentration of treatment on alternate days to reduce risk of hyperpigmentation - Lower threshold to switching to oral antibiotics if intolerance or side effects |
| Norway^15^ | **Comedonal**: Adapalene OR azelaic acid  **Mild papulopustular**: topical adapalene 0.1% and BPO 2.5% OR tretinoin 0.25mg/g and clindamycin 10mg/g  **Moderate papulopustular**: Topical adapalene 0.1% and BPO 2.5% OR adapalene 0.3% and BPO 2.5% OR tretinoin 0.25mg/g and clindamycin 10mg/g | **Mild papulopustular**: adapalene, azelaic acid or BPO gel in mono- or combination therapy  **Moderate papulopustular**: lymecycline 300mg x 1-2 OR doxycycline 100-200mg x 1-2 OR erythromycin is first choice in pregnant women | Moderate papulopustular acne | 6 – 12 weeks | Consider maintenance therapy for “young patients and if family history of prolonged and / or severe acne”. Consider either retinoids or azelaic acid. An example would be adapalene and BPO, a few days per week. | Mainly dermatologist/ specialist in skin diseases | - No specific dietary guidance, but recognition that dietary plays a role | - Advise treatment should be gradually stepped up over 2-4 weeks until daily use |
| Singapore^16^ | **Mild comedonal**: topical adapalene, topical tretinoin or topical isotretinoin.  **Mild papulopustular**: -topical clindamycin with BPO OR adapalene with BPO fixed combination products OR  topical antibiotic and BPO and topical retinoid/azelaic acid OR topical retinoid and BPO.  **Moderate papulopustular**: clindamycin-BPO OR adapalene-BPO xed combination products OR oral antibiotics and topical retinoid and BPO OR oral antibiotics and topical adapalene-BPO xed combination product OR oral antibiotics and topical azelaic acid and BPO | **Mild comedonal**: topical BPO or azelaic acid. **Moderate papulopustular**: (options for women) oral anti-androgen and topical retinoid/ azelaic acid with or without BPO. | Moderate-to-severe papulopustular acne | 6 weeks-4 months | Retinoids are recommended for maintenance | Dermatologist | - “A low GI diet is encouraged for patients with acne” - Chocolate, milk and whey correlated with acne - Insufficient evidence for withdrawal of oily or fatty foods | - Apply BPO in morning, and topical retinoid at night. Avoid simultaneous application, but topical fixed-combination products enable once daily application. - A low starting BPO concentration is recommended to reduce risk of irritation |
| UK^17^ | **For any severity of acne**: topical adapalene with topical BPO OR topical tretinoin with topical clindamycin  **For mild to moderate acne:** topical BPO with topical clindamycin  **For moderate to severe acne:** topical adapalene and topical BPO with oral antibiotic (lymecycline or doxycycline) | M**ild to moderate**: offer alternative recommended therapy  M**oderate to severe**: if initial therapy did not include an oral antibiotic, then start a recommended oral antibiotic.  If initial therapy involved an oral antibiotic, then consider dermatology referral | Moderate or severe acne.  "Oral component may be effective in treating affected areas that are difficult to reach with topical treatment (such as the back)." | 6 weeks – 3 months, but 6 months only in “exceptional circumstances” | Maintenance treatment offered if required: either combination of topical BPO and adapalene or a single topical agent (adapalene, azelaic acid, or BPO) | No information given | - Some evidence to support low GI diet, but risks of eating disorder. - Public Health England advises healthy, balanced diet. | - Initially use alternate-day or short-contact applications (e.g. wash off after an hour), then can progress to standard application |

BPO refers to benzoyl peroxide, GI refers to Glycaemic Index.

Agents with retinoid activity, such as adapalene, are included as retinoids in this table.

*Complex guideline – recommendations simplified for the purpose of this table.
